# Radiotherapy in breast cancer brain metastases: the impact of time interval and disease dynamics when breast cancer seeds to the brain

**DOI:** 10.1007/s00066-025-02378-z

**Published:** 2025-03-07

**Authors:** Katharina Hintelmann, Schohla Wahaj, Marvin Henze, Elena Laakmann, Volkmar Müller, David Krug, Tobias Gauer, Cordula Petersen

**Affiliations:** 1 Department of Radiotherapy and Radiation Oncology, University Medical Center Hamburg-Eppendorf, Martinistr. 52, 20246 Hamburg, Germany; 2 Department of Gynecology, University Medical Center Hamburg-Eppendorf, Martinistr. 52, 20246 Hamburg, Germany

**Keywords:** Prognosis, Whole-brain radiotherapy, Life expectancy, Stereotactic radiotherapy, Initial brain metastasis velocity

## Abstract

**Purpose:**

The initial brain metastasis velocity (iBMV) is a prognostic metric introduced for patients receiving stereotactic radiosurgery (SRS) for brain metastases (BM), reflecting intracranial disease dynamics. This study aimed to assess the applicability of iBMV in a mixed cohort of breast cancer brain metastases (BCBM) patients treated with SRS/fractionated stereotactic radiotherapy (FSRT) and whole-brain radiotherapy (WBRT). Considering disease dynamics, we analyzed the role of biological subtypes in determining the time interval between initial diagnosis and the occurrence of BM.

**Methods:**

We conducted a retrospective, single center cohort study of 126 BCBM patients who received radiotherapy to the brain (SRS/FSRT and WBRT) between 01/2013 and 12/2020. Statistical endpoints included iBMV, time interval between initial diagnosis and the occurrence of BM analyzed per biological subtype, and overall survival (OS).

**Results:**

Median iBMV was 0.48 BM/year. The iBMV independently predicted for mortality in the multivariate model after accounting for WBRT (hazard ratio [HR] = 1.21; 95% confidence interval [CI] 1.04–1.41; *p* = 0.012). The biologic subtype significantly influenced the time interval between initial diagnosis of breast cancer and occurrence of BM. In a multivariate model, the Karnofsky performance status and HER2 status were strongest predictors of overall survival (HR = 2.60; 95% CI 1.60–4.22; *p* < 0.001 and HR = 2.26; 95% CI 1.34–3.84; *p* = 0.002, respectively).

**Conclusion:**

iBMV correlates with overall survival, regardless of whether WBRT was used as part of local treatment. The biological subtype has a profound impact on prognosis and kinetics of BCBM.

## Introduction

Evolving treatment options for metastatic breast cancer extended the life expectancy, but also led to an increasing incidence of breast cancer brain metastases (BCBM), representing a significant cause of morbidity and mortality. Symptomatic BCBM occur in 15–20% of metastatic breast cancer patients depending on the presence of different tumor characteristics [1].

Over the past decade, radiotherapeutic treatment options for brain metastases (BM) have become increasingly complex. Trials comparing stereotactic radiotherapy (SRS) and whole-brain radiotherapy (WBRT) for up to four metastases have repeatedly demonstrated similar overall survival (OS), despite higher distant brain failure rates with SRS [2, 3]. Subsequent trials have demonstrated that SRS is noninferior to WBRT in terms of OS for patients with up to ten brain metastases, resulting in an expanded indication for SRS, where WBRT was previously the gold standard [4]. WBRT still remains a suitable option in cases with multiple BM, with prospective trials showing approximately 60% complete or partial responses and symptom palliation in about half of the patients [5]. As length of survival after BM diagnosis is increasing [6], prevention of late side effects becomes more important and cognitive dysfunctions after WBRT may impair the patients’ quality of life [7]. The recently published guideline of the DEGRO (German Society of Radiation Oncology) recommends SRS or fractionated stereotactic radiotherapy (FSRT) for a limited number [1–4] BCBM and conditionally also for 5–10 oligo-brain metastases. For multiple, especially symptomatic BCBM, WBRT is recommended [8]. However, with individual patient’s condition, comorbidities, and preferences, the radiotherapeutic approach remains personalized.

Prognostic scores may aid individual therapy decisions. One of the most widely recognized scoring system for BM is the Diagnosis-Specific Graded Prognostic Assessment (DS-GPA), which provides prognosis estimates specifically for BCBM [6]. For breast cancer, the profound impact of biological subtypes on prognosis, metastatic sites, and the time interval between initial diagnosis and the occurrence of metastasis is well-documented. Different subtypes can significantly influence the disease’s progression and treatment response, leading to a wide range of individual disease trajectories [9, 10]. Given this variability, the intracranial dynamics of the disease must be carefully considered when discussing the optimal radiotherapy (RT) modality. This aspect is not adequately reflected by the DS-GPA, which was developed as a general clinical prognostic tool and is not specifically tailored for radiotherapeutic purposes. Therefore, while the DS-GPA provides valuable prognostic information, it may not fully capture the nuances required for making precise decisions on the type of RT. The initial brain metastasis velocity (iBMV) is a metric first introduced in 2017 by Soike et al. It accounts for the number of BM at the time of first SRS divided by time since the initial primary cancer diagnosis and can reflect the intracranial dynamics. It correlates with the development of new brain metastases after initial SRS and OS and could therefore serve as a prognostic metric to risk stratify patients for WBRT or SRS/FSRT at first presentation of BM [11].

The aim of our retrospective analysis was to evaluate the applicability of iBMV in a BCBM cohort treated with different RT modalities (SRS/FSRT and WBRT). To our knowledge, no previous study has analyzed iBMV in BCBM patients including WBRT, as this novel index was developed and validated in SRS cohorts. Given the rationale that iBMV could aid in the initial choice of BM management, we analyzed whether the index remains independent of the subsequent RT modality.

Additionally, we sought to identify factors associated with time interval (TI) of BM occurrence and OS with BCBM. This knowledge can support a patient-centered treatment decision that considers individual patient and disease factors, as well as the risks of distant brain failure and potential side effects.

## Materials and methods

### Study design and patient population

We conducted a single-center cohort study. Eligible patients had histologically confirmed BCBM and received at least one course of RT to the brain between 2013 and 2020 at the University Medical Center Hamburg-Eppendorf. In detail, inclusion criteria were intracerebral metastases from no other primary tumor than breast cancer, at least one completed RT treatment for parenchymal BM, and at least one follow-up after RT. A total of 126 patients out of 179 patients screened met the inclusion criteria.

Demographic data including age, sex, date of initial diagnosis of primary tumor, stage and biological subtype of primary tumor, date of BCBM diagnosis, number and location of BCBM, Karnofsky performance status (KPS) at time of initial RT, extracranial disease, and data on BM treatment (RT and surgery) were collected from electronic medical records. Survival status and follow-up data were collected until June 2023.

Biological subtypes were defined as human epidermal growth factor receptor 2 (HER2)-positive, triple negative breast cancer (TNBC; estrogen [ER]- and progesterone [PR]-negative, and HER2-negative) and hormone receptor-positive (ER- and/or PR-positive, HER2-negative) by the primary breast cancer histology.

The number of metastases was counted up to 15 lesions and patients with ≥ 15 metastases were labelled ‘multi-metastatic’. The date of initial diagnosis of BCBM was defined as date of first presentation of BM on medical imaging (CT or MRI). Synchronous BM was defined as detection of brain metastases within 6 months after initial breast cancer diagnosis. All patients with metachronous brain metastases underwent complete treatment for the primary tumor as indicated before being diagnosed with brain metastases. Data on systemic therapy were collected only for the first intracranial RT and exclusively when the therapy was administered either within 4 weeks before/after or during the radiation treatment. DS-GPA score was calculated if data on KPS, age, subtype, extracranial metastases, and number of BCBM were available. Patients’ characteristics are summarized in Table 1A.

**Table 1:** Patient characteristics (at the initial diagnosis of brain metastases [BM], except for any second value after a slash, which represents the count after entire follow-up)

***A***	***All n*** ***=*** ***126***	***Median (IQR) or number (n)***
	Age	57.3 years (47.9–67.9)
	KPS	80% (70–90)
	*BC subtype*	
	ER-positive/HER2-negative	*n* = 39 (31%)
	HER2-positve	*n* = 51 (40%)
	Triple negative	*n* = 36 (29%)
	*Number of BM (at time of BM diagnosis/overall)*	*n* *=* *47/19*
	1	*n* = 28
	2–4	*n* = 12
	4–10	*n* = 10
	11–14	*n* = 25/36
	≥ 15 Leptomeningeal disease	*n* = 2/7
	Extracerebral metastases present	*n* = 96 (76%)
	DS-GPA	2 (1.5–2.5)
	*Number of RT courses*	
	1	*n* = 72
	2	*n* = 39
	3	*n* = 9
	4	*n* = 4
	5	*n* = 2
	*Radiotherapy*	
	Initial SRS/FSRT	*n* = 68 (54%)
	Initial WBRT	*n* = 58 (46%)
	*BM surgery*	
	Before initial RT	*n* = 49
	After initial RT	*n* = 5
	*Systemic therapy (within 4 weeks before/after first intracranial RT)*	
	Conventional chemotherapy	*n* = 52
	HER2-directed therapy	*n* = 38
	Endocrine therapy	*n* = 22
	No systemic therapy	*n* = 26
***B***	***Metachronous brain metastases (TI ID–BM ≥*** ***6 months)***	***n*** ***=*** ***92 (73%)***
	TI ID–BM	46 months (23–89)
	TI BM–death/censored	13 months (6–30.5)
	iBMV score	0.48 BM/year (0.21–1.22)

*IQR* Interquartile range (x–x), % percentage, *KPS* Karnofsky performance status, *BC* breast cancer, *BM* brain metastases, *RT* radiotherapy, *SRS* stereotactic radiosurgery, *FSRT* fractionated stereotactic radiotherapy, *WBRT* whole-brain radiotherapy, *DS-GPA* Diagnosis-specific Graded Prognostic Assessment, *iBMV* initial brain metastasis velocity, *ER* estrogen receptor, *ID* initial diagnosis, *TI* time interval, *HER2*human epidermal growth factor receptor 2

Ethical approval was waived by the local ethics committee (WF-144/20).

### Initial brain metastasis velocity

The iBMV was calculated by dividing the number of BM (at first diagnosis of BM) by the time interval (in units of years) since the initial diagnosis (ID) of breast cancer.

The iBMV was only calculated for the cohort with countable metachronous BM, defined as a minimum of 6 months between initial diagnosis of breast cancer and first diagnosis of BCBM and up to 15 distinguishable metastases on the patients’ MRI (*n* = 92, Table 1B). For instance, if a patient was diagnosed with breast cancer 4 years before the presentation of two BM, iBMV would be 0.5 BM/year. BM were counted on gadolinium-enhanced MR images and only cases with clearly distinguishable metastases were included.

$$\mathbf{iBMV}=\frac{\textit{\textbf{number}}\,\boldsymbol{of}\boldsymbol{BM}\left(\boldsymbol{at}\,\textit{\textbf{first}}\,\textit{\textbf{diagnosis}}\,\boldsymbol{of}\boldsymbol{BM}\right)}{\boldsymbol{time}\,\textit{\textbf{since}}\,\boldsymbol{ID}\left(\textit{\textbf{years}}\right)}$$

### Statistical analysis

Descriptive statistics were used to give an overview of the study cohort. OS was analyzed from date of first RT for BCBM to death/last follow-up, with collection of survival data until 06/2023. OS was analyzed using the Kaplan–Meier method. Groups were compared based on clinical and treatment-related variables using the log-rank test. All tests were two-sided, and a statistic level of significance was defined as *p*-value < 0.05.

To control for confounders in the univariable analyzes, multivariable Cox regression model was used. Covariables were chosen based on their assumed clinical influence and entered in a single step.

WBRT was included in the multivariate model to assess whether the correlation with OS remained significant, even when considering RT beyond SRS. Data were analyzed using SPSS (version 29, SPSS Inc. IBM, Armonk, NY, USA). Graphs were plotted using matplotlib library in python.

## Results

Clinical data from 126 patients with BCBM who received RT to the brain were analyzed. Median follow-up was 44 months (95% CI 24.9–63.1). Patients’ characteristics are shown in Table 1. The median age at first RT was 57.3 years (interquartile range [IQR] 47.9–67.9) and median KPS at initial BM diagnosis was 80% (IQR 70–90). Patients had mostly one or two BM-directed RT treatment courses (median 1.0; IQR 1–2), with a maximum of five courses. Across all therapy sessions, 65.1% (*n* = 82/126) patients remained ‘non-multi metastatic’ (≤ 15 BM). The median was 3 BM in total during follow-up (IQR 2–7), while 19 patients (15.1%) had only a single BM for the whole follow-up period and 47 patients presented with a single brain metastasis at initial diagnosis of BCBM. In all, 28.8% had multiple BM (> 15 BM; *n* = 36/126), and 6% (*n* = 7/126) developed leptomeningeal disease. A total of 392 countable BCBM were analyzed, including 42 locally recurrent metastases.

**Fig. 1:**
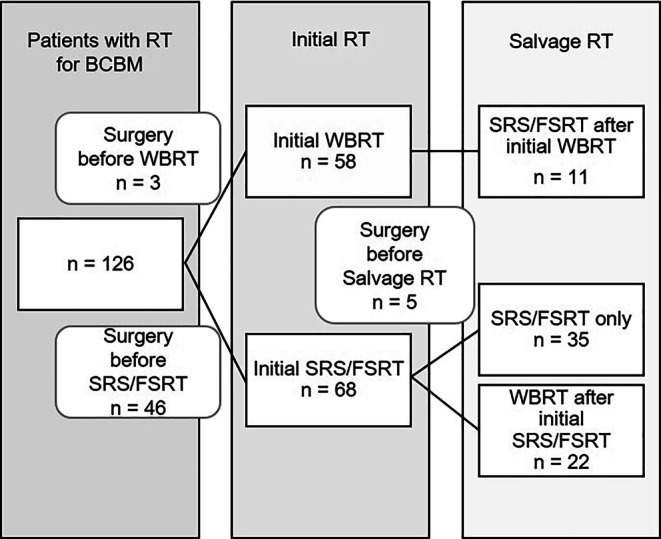
Overview of radiotherapy (RT) courses after initial diagnosis of breast cancer brain metastases (BCBM). SRS stereotactic radiosurgery, FSRT fractionated stereotactic radiotherapy, WBRT whole-brain radiotherapy

The multidisciplinary treatment of BCBM with repeated courses of local RT leads to complex medical histories. Figure 1 illustrates an overview of RT courses after diagnosis of BCBM. The type of initial RT was approximately equally distributed, with 46% WBRT (*n* = 58/126) and 54% (*n* = 68/126) SRS/FSRT. Initial metastasis surgery was mainly performed before the first SRS/FSRT with 71% (*n* = 46) of all surgeries during the follow-up period at this point. Salvage RT due to first BM progression after initial WBRT was only by SRS/FSRT. A total of 22 patients received salvage WBRT after initial SRS/FSRT. Of note, 27.8% of patients (*n* = 35) received no WBRT during the follow-up and could be treated with one or more salvage SRS/FSRT courses after BCBM progression.

**Fig. 2:**
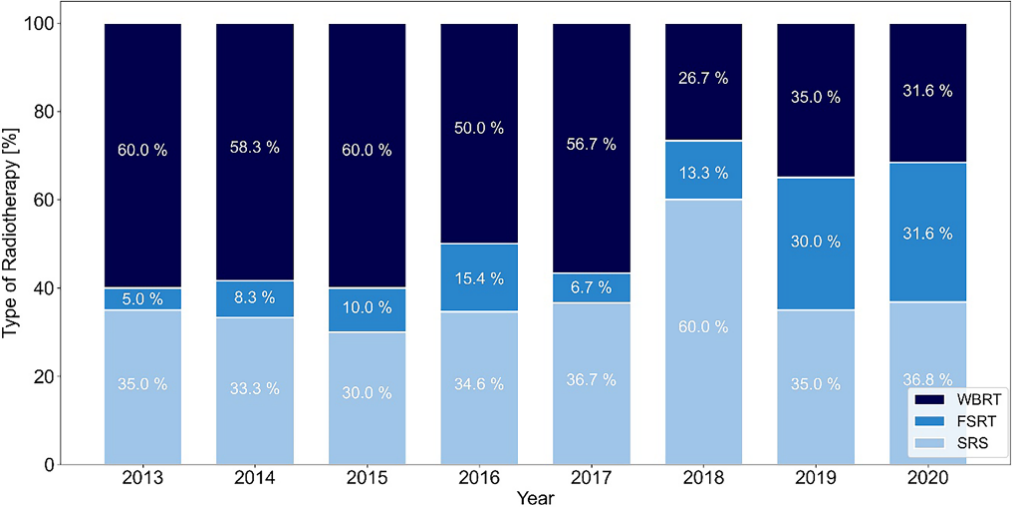
Type of local radiotherapy (RT) for breast cancer brain metastases (BCBM) per year (2013–2020). SRS stereotactic radiosurgery, FSRT fractionated stereotactic radiotherapy, WBRT whole-brain radiotherapy

Fractionation of WBRT was mostly 10 × 3 Gy. For SRS, doses between 18 Gy and up to 24 Gy were used. FSRT was mostly delivered in 3 × 9 Gy, 5 × 6–7 Gy, and 7 × 5 Gy, with the use of the more fractionated concepts mainly for cavity-RT after metastasis resection. Figure 2 shows the relative use of the different RT techniques for the years 2013–2020. From 2013–2015, WBRT was the most common type of RT for BCBM with approximately 60% declining to about 30% in 2020. Since 2018, stereotactic approaches have become more frequent than WBRT and especially the use of FSRT increased from 5% in 2013 to 32% in 2020.

At the time of the first BCBM diagnosis, 76% of patients presented with extracerebral metastases. Thus, only 26 patients did not receive systemic therapy within 4 weeks before or after their first intracranial RT. A total of 89 patients received some form of systemic therapy, with conventional chemotherapy being the most common (*n* = 52), followed by HER2-targeted therapies (*n* = 38) and endocrine therapy (*n* = 22). Trastuzumab was the most frequently used HER2-targeted therapy, while paclitaxel was the most commonly administered chemotherapy, followed by capecitabine.

### Initial brain metastasis velocity and the time interval since initial diagnosis

The median time interval between initial BC diagnosis and diagnosis of BCBM in this cohort was 46 months (IQR 23–89). A total of 21 patients (16.6%) showed a late occurrence of BCBM (TI ≥ 10 years). A shorter TI of < 5 years (*n* = 80; 63.5%) was not associated with a worse OS.

**Fig. 3:**
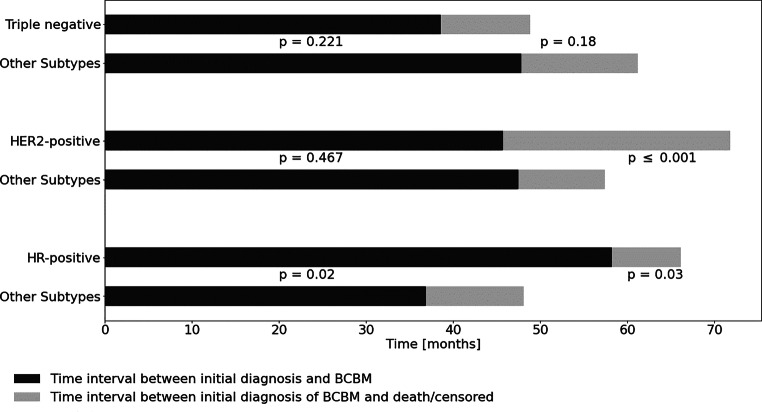
Time interval between initial diagnosis, diagnosis of breast cancer brain metastases (BCBM) and death/censored analyzed according to biological subtypes. Hormone receptor-positive (HR): estrogen receptor-positive (ER) and ± progesterone receptor-positive (PR). Human epidermal growth factor receptor 2 (HER2)-positive: HER2-overexpression and HR-negative. Triple negative: HR-negative and HER2-negative. Other subtypes: All cases not included in the subtype-specific upper bar are summarized in the lower bar for comparison. *P*-values were calculated for the differences in the logarithmic mean time interval for each subgroup using a two-sided t‑test with the left *p*-values corresponding to the black bars and the right *p*-values to the grey bars

BM from hormone receptor-positive BC were significantly associated with a longer TI between ID and diagnosis of BCBM, whereas triple-negative BC shows a tendency towards early occurrence of BM. Patients with BM from HER2-positive BC had a remarkably longer survival after the first occurrence of BM (Fig. 3).

Of note, dissimilar to the other presented results, the iBMV was only calculated for the cohort with countable metachronous BM, defined as a minimum of 6 months between the initial diagnosis of breast cancer and the first diagnosis of BCBM, and ≤ 15 metastases countable on the patient’s MRI (see “Methods” section; *n* = 92, Table 1B). The median iBMV was 0.48 BM/year (IQR 0.21–1.22). Of these patients, 66 had 1–3 metastases, 17 had 4–10 metastases, and 9 had 11–14 metastases.

**Fig. 4:**
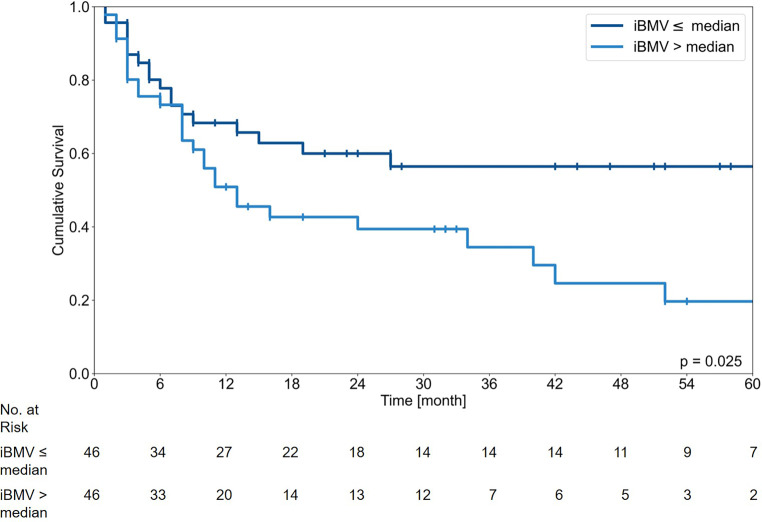
Kaplan–Meier survival curve for initial brain metastasis velocity (iBMV) ≤ or > the median of 0.48 brain metastases/year. No. Number

Risk classes by the iBMV index are not consistently defined and a threshold of two is proposed by some authors [12, 13], but as the reported iBMV for breast cancer is lower, we used the median for separation into two groups like initially published by Soike et al. [11]. In the Kaplan–Meier estimate an iBMV index above the median of 0.48 BM/year was significantly associated with a worse OS (*p* = 0.025; Fig. 4).

In the univariate Cox regression, iBMV above the median was significantly associated with a higher risk of death (*p* = 0.031; HR 1.92 [95% CI 1.06–3.46]) and an increase of the iBMV index by 1.0 BM/year was associated with a 1.17 increase in hazard ratio (HR; *p* = 0.011 [95% CI 1.04–1.31]; Table 2).

**Table 2:** Univariate and multivariate analysis of overall survival

Variable			Univariate HR (CI 95%) (*p*-value)		Multivariate HR (CI 95%) (*p*-value)	
	Whole cohort	iBMV cohort	Whole cohort *n* = 126	iBMV cohort *n* = 92	Whole cohort *n* = 118	iBMV cohort *n* = 89
Extracranial metastases	*Present: 96*	*Present: 66*	1.82 (1.02–3.26), *p* *=* *0.043*	1.47 (0.75–2.89)*, p* = 0.264	1.64 (0.88–3.05)*, p* = 0.117	1.23 (0.59–2.53)*, p* = 0.583
	30	26				
HER2 status	*75*	*53*	1.92 (1.19–3.11), *p* *=* *0.008*	2.27 (1.22–4.22), *p* *=* *0.009*	2.26 (1.34–3.84), *p* *=* *0.002*	3.19 (1.60–6.37), *p* *=* *<* *0.001*
	51	39				
Number of BM^a^	Per increase by 1 BM	Per increase by 1 BM	1.05 (1.01–1.09), *p* *=* *0.028*	1.01 (0.95–1.07), *p* = 0.706	1.01 (0.95–1.07)*, p* = 0.823	0.95 (0.88–1.04)*, p* = 0.263
KPS	*<* *80: 41*	*<* *80: 28*	2.60 (1.63–4.13), *p* *=* *<* *0.001*	2.53 (1.40–4.56), *p* *=* *0.003*	2.60 (1.60–4.22), *p* *=* *<* *0.001*	2.46 (1.29–4.70)*, p* *=* *0.006*
	≥ 80: 85	≥ 80: 64				
WBRT	*Yes: 76*	*Yes: 45*	2.46 (1.46–4.13), *p* *=* *<* *0.001*	2.21 (1.21–4.03), *p* *=* *0.010*	2.10 (1.03–4.29), *p* *=* *0.042*	2.15 (0.99–4.63)*, p* = 0.052
	50	47				
iBMV	Per increase by 1 BM/year	Per increase by 1 BM/year	–	1.17 (1.04–1.31), *p* *=* *0.011*	–	1.21 (1.04–1.41), *p* *=* *0.012*

Indicator variables are shown in italics

*BM* Brain metastases, *HR* Hazard ratio, *HER2*Human epidermal growth factor receptor 2, *iBMV* initial brain metastasis velocity, *KPS* Karnofsky performance status, *WBRT* whole-brain radiotherapy

^a^ Patients with leptomeningeal disease were excluded (*n* = 7, unknown *n* = 1). For analysis of the whole cohort 15 BM or more were counted as 15 BM

In comparison, TI between the ID and the diagnosis of BM and the number of BM at this time point, both had a HR of 0.99 and 1.09, respectively, in a bivariate Cox regression model. This indicates that neither the TI nor the number of BM alone has a substantial impact on OS compared to both combined in the iBMV index.

### Survival analysis

Median OS in the whole cohort was 15 months (95% CI 9.0–21.0). In univariate analysis, presence of extracranial metastases, HER2 status, KPS, number of BCBM, receiving WBRT and iBMV above the median of 0.48 BM/year were identified as significant variables affecting survival. Of note, the analysis of iBMV was done in a cohort of 92 patients as described before, and results are therefore shown in separate columns per analyzed cohort. For this subcohort, only HER2 status, KPS, receiving WBRT, and iBMV had an association with OS in univariate analysis (Table 2).

To test whether the iBMV has a significant impact on OS, we analyzed all covariables in the iBMV cohort (status of all covariables available in *n* = 89 patients) and compared results to the multivariable analysis of the other covariables in the whole cohort (status of all covariables available in *n* = 118 patients). As per multivariable analysis in the iBMV cohort, iBMV (per increase by 1 BM/year) was significantly associated with worse OS (HR = 1.21; 95% CI 1.04–1.41; *p* = 0.012). In the iBMV cohort and the whole cohort, HER2-negative BC (HR = 2.26; 95% CI 1.34–3.84; *p* = 0.002) and KPS < 80% (HR = 2.60; 95% CI 1.60–4.22; *p* = < 0.001) were significantly associated with worse OS (Table 2).

An analysis of the impact of systemic therapies (chemotherapy, HER2-targeted therapy, and endocrine therapy) administered within 4 weeks before or after the first intracranial RT on OS revealed that the prognostic value of HER2 status depends on the receipt of HER2-targeted therapy. When these covariates are included in the multivariate analysis, HER2 status was not significantly associated with OS, although the trend of other variables remained unaffected. Notably, the iBMV was still significantly associated with worse OS in the iBMV cohort (HR = 1.26, 95% CI 1.10–1.49; *p* = 0.007) in this model.

## Discussion

iBMV independently predicted OS in the multivariate model after adjusting for WBRT, with KPS and HER2 status presenting as the strongest predictors of OS. The individual variables used to calculate iBMV were not significant predictors of OS on their own; only the combined metric of iBMV demonstrated predictive value for survival.

The temporal relationship between the initial cancer diagnosis and the development of BM is not captured by the established DS-GPA, highlighting the potential of iBMV as a valuable additional prognostic marker. However, iBMV has the limitation, i.e., it cannot be applied to patients with synchronous BM presentation. Despite the high incidence of BM in metastatic breast cancer—particularly in HER2-positive and triple-negative subtype—there is no evidence supporting the screening of asymptomatic patients [14]. This creates uncertainty when calculating iBMV, because the asymptomatic period of BM is undefined. This is particularly relevant when applying iBMV to patients with incidentally discovered brain metastases or those identified through screening in asymptomatic individuals. Synchronous presentation with BM is less common in breast cancer compared to other tumor types, such as lung cancer or melanoma [15]. Most BCBM occur metachronously during follow-up or palliative treatment for extracranial metastases [16]. Prior treatment regimens can influence the disease course and the clonal evolution of tumor cells, shaping their biological characteristics. Malignant cells may remain dormant behind the blood–brain barrier before causing symptoms [17]. Therefore, synchronous and metachronous central nervous system (CNS) metastases may represent distinct entities with different biological behaviors. Furthermore, the treatment approach for patients with synchronous metastases differs, as the focus shifts from treating the breast tumor to controlling metastatic lesions. These potential biological and clinical differences should be carefully considered when applying iBMV. With a median of 46 months between primary tumor diagnosis and the diagnosis of BM, most patients in this cohort experienced a relatively long BM-free interval. The shortest TI observed in the iBMV cohort was 11 months. In our study, all patients classified as having metachronous metastases had completed their initial curative-intent treatment for the primary tumor.

The threshold of 0.48 BM/year, based on the median iBMV in our cohort, was chosen to stratify patients for analysis. However, this value is cohort specific and lacks broader standardization, which may complicate external validation and the comparability of findings across studies. This median split was also employed by Soike et al., who reported a median of 0.79 BM/year in their cohort with metachronous BM and used this value for risk stratification. Consistent with our data, they identified a relatively low iBMV of 0.44 (IQR 0.21–0.90) for breast cancer compared to 1.43 BM/year (IQR 0.72–2.78) for lung cancer [11]. Furthermore, Yamamoto et al. used an iBMV score of 2.0 BM/year as a cut-off, which was close to the median of 2.07 found in their cohort of different primary cancer types, but they also reported a different distribution within the risk groups, with 60% of patients with non-small cell lung cancer (NSCLC) having iBMV > 2.0 BM/year and in contrast only 37% of patients with breast cancer [18]. This highlights the challenge of establishing a uniform threshold across different cohorts with varying primary tumor histologies.

Data on the validity of iBMV across different local BM therapy approaches are sparse. Kimura et al. have shown the applicability for patients with NSCLC BM treated with surgery, systemic therapy, WBRT, and SRS [13]. In addition to predicting OS, iBMV has been shown to correlate with brain metastasis velocity (BMV) [11, 18]. BMV is defined as the cumulative number of new brain metastases that develop over time since the first SRS, and this metric correlates with OS, neurologic death, and the need for salvage WBRT following initial distant brain failure after upfront SRS alone [19]. However, since assessing the correlation with BMV was not the primary objective of our study, and BMV by definition was only applicable in 20 patients of our cohort, no definitive conclusions could be drawn regarding the correlation of iBMV with BMV. This endpoint clearly warrants further studies, as it reflects the intracranial disease dynamic after diagnosis and treatment of BM.

The decision between stereotactic approaches (SRS/FSRT) and WBRT remains a complex, individualized process influenced by numerous factors, and iBMV alone cannot fully address this decision-making challenge. It is important to note that, while an association between iBMV and OS, as well as BMV has been reported [11, 18], Ho et al. demonstrated a significant association with brain progression-free survival (PFS), but the same study also found that iBMV was not predictive of the discrimination between intracranial local or distant relapse [20]. Thus, iBMV may provide a general indication of whether a patient requires more aggressive treatment (such as upfront WBRT) to address rapid disease progression. Conversely, patients with a low iBMV and favorable prognostic indicators may benefit from stereotactic approaches that minimize long-term neurocognitive side effects. However, it cannot definitively determine the choice between stereotactic therapy and WBRT, but offers a valuable easily calculable metric that could be integrated into routine evaluation of patients presenting with BCBM for the first time.

With a median OS of 15 months in our cohort, the prognosis for patients with BCBM remains poor compared to other groups, such as patients with bone-only metastasis, who have a relatively favorable survival, averaging between 24 and 65 months [21]. However, it is noteworthy that 10 patients in our cohort achieved exceptionally long survival, living more than 5 years after their first RT of BCBM. Our data did not reveal a direct correlation between OS and the time interval between the initial BC diagnosis and the occurrence of BM. Interestingly, Michel et al. [22] reported that for BCBM patients undergoing metastasis surgery, the occurrence of BCBM within 5 years after ID was independently associated with a worse prognosis after BM surgery. It is well established that the biological subtype influences the incidence, kinetics, and prognosis of BCBM [23, 24]. For our data, the longest TI between primary tumor diagnosis and BM was observed in HR-positive, HER2-negative BC. The HER2 status has also frequently been shown to have strong prognostic significance, consistent with our data [25]. Cagney et al. further reported that the biological subtype influences intracranial recurrence patterns following brain-directed RT for BCBM, with HER2-positive BCBM exhibiting more local progression, while the triple-negative subtype was associated with distant progression [26]. In addition, it has been described that the application of HER2-targeted therapy after or prior to the BM diagnosis is associated with better OS [27]. In our study, data on concurrent or simultaneous HER2-targeted therapy were incomplete and therefore we could not fully address this point in our results; however, by including data on systemic therapies administered at the time of the first intracranial treatment, we were able to demonstrate that the prognostic value of HER2 status is not independent of HER2-targeted therapies, as would be expected. In a large breast cancer cohort published by Yamamoto et al., the correlation between iBMV and OS was demonstrated across all biological subtypes, except for HR-positive breast cancer, when comparing an iBMV greater than 2 versus an iBMV of 2 or below [12].

Our study is limited by its retrospective design and incomplete data on systemic therapy, which may introduce selection bias inherent to this study type and potential information bias. The exclusion of patients with synchronous metastases and those with more than 15 brain metastases from iBMV cohort further contributes to the potential for selection bias. Additionally, temporal changes cannot be fully captured, which is particularly relevant concerning advancements in systemic therapies with CNS activity. Most systemic therapies with CNS activity have been approved for HER2-positive breast cancer and within the last 5 years, and this should be considered when interpreting the generalizability of our findings to current patient cohorts. Furthermore, the relatively small cohort size limited our ability to analyze iBMV across different biological subtypes and to investigate its correlation with BMV, which could have provided deeper insights into disease dynamics beyond overall survival. Nevertheless, our findings contribute valuable knowledge regarding variables affecting disease progression and prognosis, which are crucial for personalized treatment decisions. Future research should assess the impact of systemic treatments on iBMV and examine how iBMV correlates with time to new brain metastases across various radiotherapy modalities. In summary, our study supports iBMV as a predictor of OS in BCBM patients and highlights the need for further research to explore its predictive potential.

## Conclusion

Initial brain metastasis velocity (iBMV) correlates with overall survival (OS) in a mixed breast cancer brain metastases (BCBM) cohort treated with stereotactic radiosurgery/fractionated stereotactic radiotherapy (SRS/FSRT) and whole-brain radiotherapy (WBRT), indicating its potential to predict disease dynamics. Further studies and validation should include systemic treatments and evaluate the correlation with time to new brain metastases for each radiotherapy modality. The biological subtype significantly influences the prognosis and kinetics of BCBM.

## Abbreviations

BC: breast cancer
BCBM: breast cancer brain metastases
BM: brain metastases
BMV: brain metastasis velocity
DS-GPA: Diagnosis-specific Graded Prognostic Assessment
ER: estrogen receptor
FSRT: fractionated stereotactic radiotherapy
HER2: human epidermal growth factor receptor 2
HR: hormone receptor-positive
iBMV: initial brain metastasis velocity
ID: initial diagnosis
KPS: Karnofsky performance status
OS: overall survival
PR: progesterone receptor
RT: radiotherapy
SRS: stereotactic radiosurgery
TI: time interval
TNBC: triple negative breast cancer
WBRT: whole-brain radiotherapy
